# Female Disparity in Referral to Cardiac Diagnostication and Invasive Treatment

**DOI:** 10.3390/medicina62010144

**Published:** 2026-01-10

**Authors:** Rajesh Bhavsar, Leif Thuesen, Carl-Johan Jakobsen

**Affiliations:** 1 Department of Anaesthesiology and Intensive Care, University Hospital of Southern Denmark, 6200 Aabenraa, Denmark; rbhavsar@health.sdu.dk; 2 Department of Regional Health Research, University of Southern Denmark, 5230 Odense, Denmark; 3 Department of Cardiology, Aalborg University Hospital, 9000 Aalborg, Denmark; thuesenleif@gmail.com; 4 Department of Anaesthesiology, Heart, Lung and Vessel, Aarhus University Hospital, 8200 Aarhus N, Denmark

**Keywords:** cardiovascular diseases, coronary artery disease, healthcare disparities, ischemic heart disease, sex differences, invasive cardiac procedures

## Abstract

*Background and Objectives:* Despite huge reductions in the incidence and mortality of cardiovascular disease (CVD) during the last decades, ischemic heart disease (IHD) is globally still a leading cause of death. Although females experience higher mortality, the clinical IHD guidelines do not distinguish between sexes, and despite added diagnostic procedures after introduction of computed coronary arteriography (CTA) the differences remain. This study aimed to describe and evaluate the effect, outcomes and sex disparities of the large number of diagnostic procedures not leading to invasive treatments. *Materials and Methods:* The study included 274,617 first-entry patients submitted to invasive coronary arteriography (ICA) or CTA 2000–2020, from the mandatory Western Denmark Heart Registry. Mortality was evaluated with Kaplan–Meier curves and further compared to background population. *Results:* Females constituted 34.1% of all first-entry diagnostic procedures but only 25.5% of those who subsequently underwent invasive treatment, demonstrating a substantially lower treatment rate compared to males. All-cause 10-year mortality was higher in females after treatment 1.26 (1.23–1.30) but lower in the non-treated patients 0.71 (0.67–0.72) at all time points. Comparing to the background population, all non-treated patients revealed lower mortality in all indications, except valves. *Conclusions:* Despite being referred for coronary diagnostication according to their CVD prevalence, females received less invasive treatments than males and presented with substantially higher mortality after invasive treatments. In variance, non-invasive treated females demonstrated significantly better survival than men both in intra-study comparisons and in assessment with background population mortality.

## 1. Introduction

Despite the last decades’ dramatic reductions in the incidence and mortality of cardiovascular disease (CVD) particularly in developed countries [1,2], ischemic heart disease (IHD) is still the leading cause of global mortality and a major contributor to disability in both genders [3]. CVD accounts for over 30% of worldwide death, with IHD being the leading cause, accounting for 16% in 2019 [1,2]. Notwithstanding, age-standardized IHD prevalence, incidence, and mortality has declined from 1990 to 2019 regardless of sex [4,5], with a similar decrease in Denmark between 1970 and 2015 [6]. Likely contributors to the positive development are lifestyle changes, improved preventative strategies, enhanced surveillance, and improved medical therapy together with quicker and more advanced diagnostics. Additionally, enhanced treatment of heart failure and augmented coronary revascularization, especially in acute coronary syndrome, may have contributed further to ameliorate the IHD health problem [6,7,8].

Despite uniform efforts and contrary to expectations and the fact that overall female mortality has declined more than that of males, females still experience higher IHD-related mortality than males [4,9,10]. This disparity is believed to be caused by factors like older age at referral, a higher degree of atypical symptoms, and less frequent treatment with evidence-based therapy among females [10,11,12]. Further, females often experience a poorer prognosis after acute cardiovascular events, indicating a higher overall cardiovascular risk profile [13]. Reports claim that CVD risk in females is often underestimated, perhaps due to the perception that women are protected against CVD and consequently experience a lower rate of diagnostic angiograms and interventional procedures as compared to men [14].

The diagnostic algorithms in patients with suspected chronic coronary syndrome (CCS) have been changed considerably by the introduction of computed tomography angiography (CTA) as a standard first-entry method around 2007/2008. The purpose was to reduce diagnostic invasive coronary angiography (ICA) procedures in patients unlikely to require revascularization [15,16,17]. However, data from the Western Denmark Heart Registry (WDHR) show an overwhelming increase in CTA procedures, without a corresponding increase in either the number of PCI or cardiac surgery procedures. Instead, CTA appears to have expanded into a broader range of indications, beyond its original gatekeeping role.

Using WDHR data from 2000 to 2020, this study aimed to describe diagnostic trends, clinical outcomes and sex-related differences associated with the large proportion of diagnostic procedures that did not lead to subsequent invasive treatment. Furthermore, we compared observed mortality in diagnostic, treatment, and sex subgroups with the age- and sex-matched expected mortality of the general Danish population during the same period.

## 2. Material and Methods

The data were obtained from WDHR, established in 1999 and fully functional in 2000, containing information of all adult patients from the Western part of Denmark undergoing diagnostic or interventional cardiac procedures. WDHR is mandatory and initially comprised the diagnostic procedure ICA and the interventional treatments of percutaneous coronary intervention (PCI) and cardiac surgery. Computed tomography angiography (CTA) and transcatheter aortic valve replacement (TAVR) were added in 2008.

WDHR data are collected and registered prospectively, and encompass detailed patient-, risk-, procedure-, and care-related data. The database is an integral part of daily clinical practice [18]. Further, WDHR is known for accuracy and completeness warranted by inborn data validation at entry, random spot checks and systematic validation procedures [19]. All data related to this study have been obligatory since 2006. Earlier data fields were considered 0 or negative if any other data was registered on the specific formulas.

All Danish citizens have a unique civil personal registration (CPR) number assigned at birth or immigration and kept throughout life, enabling cross-linking between different health and civil registries, ensuring feasibility to conduct large, population-based studies with relevant outcome follow-up on all procedures and treatments [20,21,22].

### 2.1. Ethics and Permission

The study data handling was approved and registered by the Danish Data Protection Agency (1-16-02-455-21). Written consent is not required for registry-based studies according to Danish legislation. The agency’s directions for data handling were met.

### 2.2. Study Population

**Figure 1:**
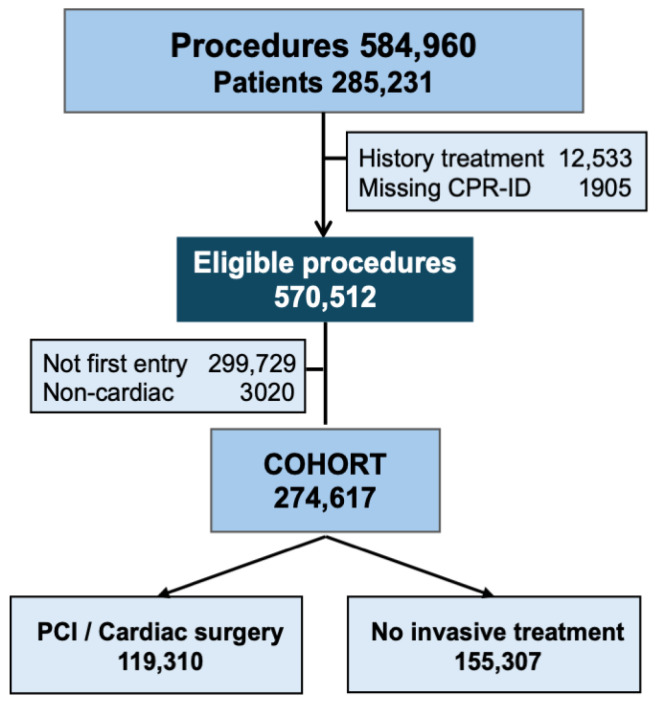
Screened procedures obtained from Western Denmark Heart Registry 2000–2020 and the exclusions. CPR = civil personal registration; non-cardiac = a mixture of research, projects, screening without symptoms and before non-cardiac surgery; PCI = Percutaneous coronary intervention.

All diagnostic ICA (304,980) and CTA (114,324) as well as the treatments of PCI (116,045), cardiac surgery (45,677) and TAVR (3968) between 2000 and 2020 were considered for eligibility (Figure 1). Primary exclusions were patients without a valid CPR and a history of invasive cardiac treatments, and secondary were all non-cardiac indications and not first-entry procedures. Thus, only the first procedure was included, in cases where the patients were undergoing multiple procedures.

Procedure indications were grouped at discretion of the attending doctor at procedure according to register indication into (1) acute coronary syndrome (ACS), (2) chronic coronary syndrome (CCS), (3) valves, (4) cardiomyopathy/heart insufficiency, (5) arrhythmias, (6) cardiogenic shock/arrest and (7) other. If there were overlapping indications, the case was allocated the most severe indication, i.e., CABG and valve was allocated to the valve group. To secure uniform indication, all were compared to the registered ICD-code; if there was divergence the indication was based on registered ICD-10 (Supplementary Table S1).

### 2.3. Outcome Parameters

The primary analysis was ICA and CTA with or without an invasive treatment procedure, i.e., PCI, cardiac surgery or TAVR, with the main focus on the non-treated patients.

The primary outcome was all-cause mortality after stipulated periods over the 20-year study period. The material was subdivided according to sex, diagnostic ICA or CTA, together with treatment procedures at points of time.

The registered consequences differed between ICA and CTA in WDHR. Therefore, the data were re-grouped into harmonised comparable groups (Supplementary Table S2). The group “no-treatment” covers both patients with normal findings and those with findings without possible treatment.

Considering differences in life expectancy between men and women, and the large change in the 20-years study period, all patients were assigned an estimated 1- and 5-year risk of death based on actual 5-year life tables (Supplementary Figure S1) acquired from Denmark Statistics [23]). These expected 1- and 5-year mortality risks were based on year of procedure, age, and sex, enabling analyses of the study’s actual mortality against the background population mortality in a 1:1 ratio.

### 2.4. Statistical Analyses

Patients were divided by age, sex, indication and procedure type for the detailed statistical analyses. Relevant grouping of patients was used where appropriate to avoid small group sizes. Categorical variables were analysed using the χ^2^-test, and continuous variables by Student’s independent *t*-test and ANOVA in normally distributed data, or Mann–Whitney test and Kruskal–Wallis tests in non-normally distributed data. Kaplan‒Meier survival curves were used to evaluate outcomes over time and to compare with the mortality of the background population. Logistic regression analyses, with robust error variance to estimate adjusted odds ratios (ORs), were used to identify risk factors with an impact on “risk” of submitting to invasive treatment and outcomes presented as OR with 95% confidence limits (CLs). The included covariates were based on comorbidity factors, indication and sex. Analyses were performed with MedCalc^®^ Software Ltd., version 23.4 (Ostend, Belgium). A probability value of <0.05 was used to define statistical significance.

## 3. Results

The selection process revealed 584,994 procedures in 285,231 patients registered in WDHR from 2000 to 2020. The primary exclusions were 1905 without valid CPR, and 12,544 with a history of invasive cardiovascular treatment or surgery. Secondary exclusions were 299,729 not first-entry cases and 3020 non-cardiac indications, isolating a cohort of 274,617 first-entry patients/procedures for analysis (Figure 1). Especially in the early period, the treatment procedures encompassed a higher fraction of the first-entry procedures. This was partly because the diagnostics were performed before WDHR had fully implemented a standardized registration of diagnostics together with other diagnostic measures like standard computerized tomography, magnetic resonance imaging and ultrasound before especially cardiac and aortic surgery and TAVR. Regarding early PCI, there may have been a registration issue, as many ICA and PCI procedures were often combined procedures with almost identical registered parameters, making it difficult to separate the timing and classify the procedures accurately.

**Figure 2:**
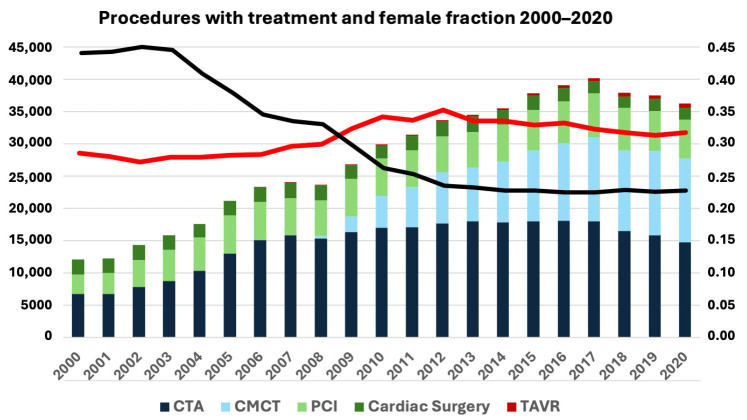
All registered procedures in Western Denmark Heart Registry 2000–2020 divided by procedure type and years together with female fraction (red line) and treatment fraction (black line).

**Figure 3:**
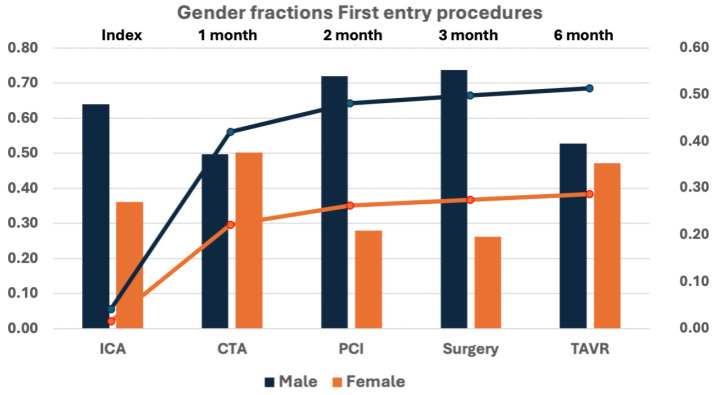
All first-entry procedures divided on procedure types with gender fractions (columns) and the accumulated number of treatment procedures (PCI, cardiac surgery, TAVR) within the first 6 months (lines). Females were significantly lower in all procedures (*p* < 0.0001; χ^2^-test). Total female diagnostic procedures were 39.8% and treatment procedures 27.1%.

The total yearly procedure number increased from 12,139 in 2000 to a maximum of 40,184 in 2017. In contrast, the treatment fraction decreased from 45.4% in 2002 to 22.9% in 2017 of all submitted procedures (Figure 2). Overall, we found an almost 5-fold increase in diagnostic procedures compared to only a 66% increase of invasive treatments. Despite the increased number of invasive treatments, the fraction of the treatment declined due to the increase in diagnostic procedures, especially in arrhythmias and CCS. The indexed number of diagnostic procedures decreased for all indications (Figure 3). Females accounted for 34.1% of all entries, with a small increase from 30.7% during the first 5-year period to 34.4% during the last 5-year period (Figure 2), and presented lower in all procedure types with 37.4% of all diagnostic procedures and 25.5% of treatments (*p* < 0.0001; χ^2^-test). Analysing first-entry procedures, the female fraction was marginally higher, increasing from 33.7% to 41.6% from the first to the last 5-year period and showing great diversity from 26.2% in cardiac surgery to 50.2% of CTA procedures.

All registered demographics (Table 1) changed over time, primarily due to the introduction of CTA late in the second period. Overall, females were marginally older (63.7 vs. 62.3), presented with a higher fraction of IHD in family (42.9% vs. 35.4%), and more often had hypertension treatment (47.3% vs. 43.3%, but without differences in statin treatment. Previous myocardial infarction (8.6% vs. 4.5%) and diabetes treatment (10.9% vs. 9.1%) were more often seen in males (Table 1).

The number of diseased coronaries differed among both sex and treatments. On average, males had 2.46 diseased coronary arteries compared to 2.15 in females and a higher average diameter stenosis as well, being 81.8% vs. 79.0%. Regarding the diagnostic-only patients, the number was marginally different with males having 2.28 diseased coronaries vs. females 1.95, and in treated groups males 2.49 vs. females 2.22 (Table 1). Concerning average diameter stenosis, the values were lower in non-treated individuals (men 71.2% and women 66.0%) than in treated individuals (men 84.1% and women 83.1% (Table 1).

**Figure 4:**
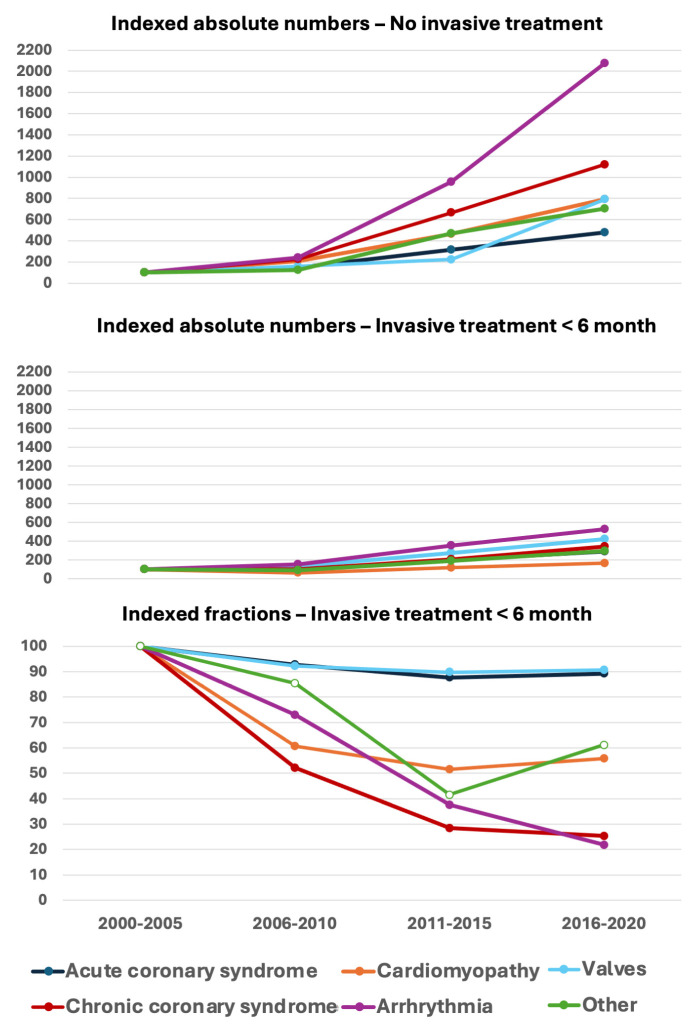
Indexed absolute numbers in diagnostic procedures without treatment (upper panel) and absolute numbers (middle panel) and indexed fraction of invasive treatment within 6 months divided by indications and time periods. The differences in both indications and periods were significantly different (*p* < 0.001; 2-way ANOVA). Despite increased number of invasive treatments, the fraction is diminished due to extreme increase in diagnostic procedures in some indications, especially arrhythmias and CCS.

The registered demographics were significantly different between the indication groups, as shown in Table 2, which also demonstrated that the fraction of females was significantly lower in all indications (*p* < 0.001; 2-way ANOVA). The sex disparity was underlined by the difference in accumulated treatments; females 28.2% vs. males 49.1% within the first 6 months after the diagnostic procedure (Figure 4). Together with later treatments (females 2.01% and males 3.01%), 78,508 (47.8%) males and 76,799 (69.6%) females were left with diagnostication only.

**Figure 5:**
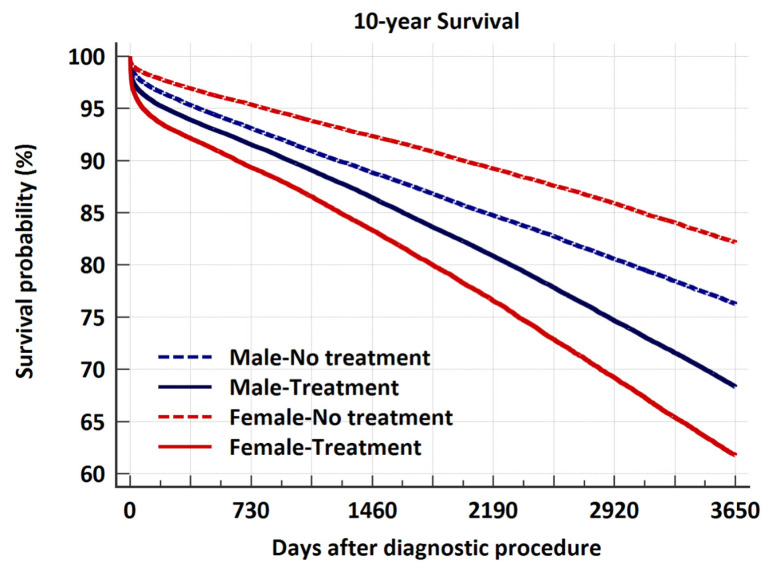
10-year survival divided on treatment within 6 months or no treatment and gender. In treatment groups, females had lower survival than males (1.26 (1.23–1.30)), while if no treatment females had better survival (0.71 (0.69–0.72)).

Females treated within 6 months after diagnostication with ICA/CTA had lower survival than males 1.26 (1.23–1.30). In contrast, we found better survival in non-treated females 0.71 (0.69–0.72) (Figure 5).

The consequences of the diagnostic procedures showed huge differences between indications. The fraction of “No further” consequence was higher in females than males (46.5% vs. 39.1%; *p* < 0.0001; χ^2^-test) and lower in the consequences “Medical” and “Invasive treatment” (Table 3). The number of patients suggested for treatment, where treatment was not carried out, was low in all indications ranging from 1.0% in valvular heart disease to 0.4% after cardiogenic shock. The absence of treatment was likely not caused by early mortality, as the 7-day mortality varied from 0.11% in CCS patients to 2.52% in ACS patients. However, in cardiogenic shock/cardiac arrest the 7-day mortality was 47.6%. The 30-day mortality ranged from 0.23% in CCS to 6.79% in valve indications (Table 4).

**Figure 6:**
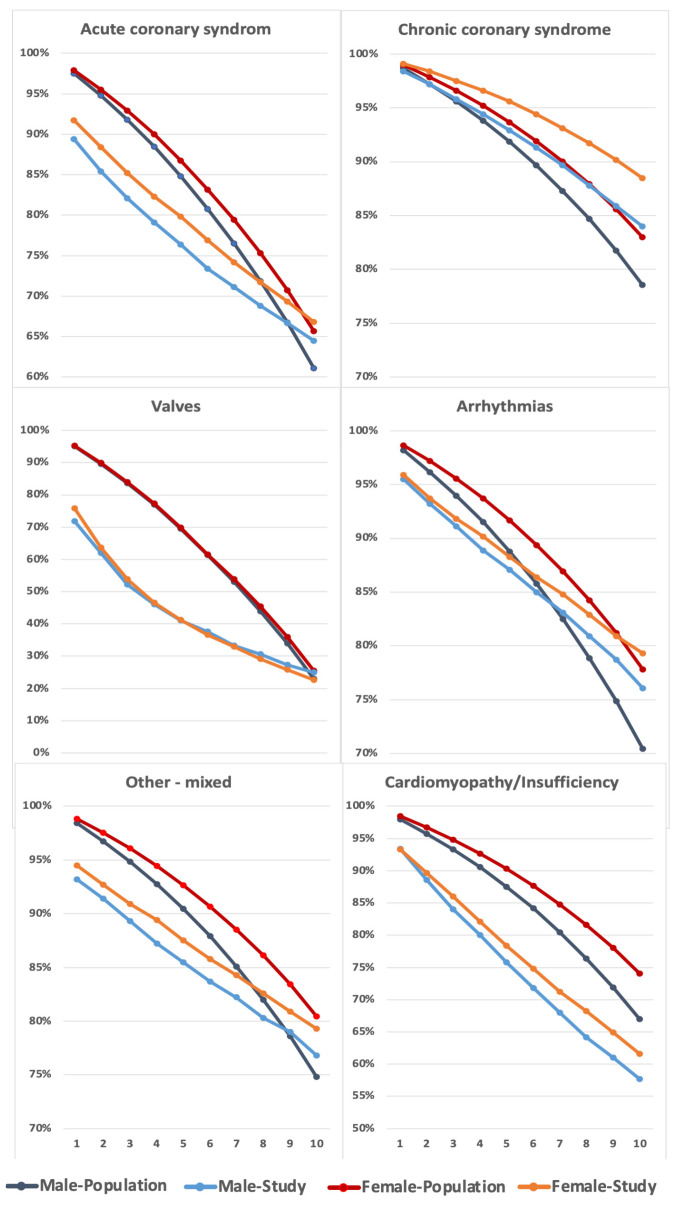
Actual and calculated population 10-year survival of all first-entry diagnostics, non-treated procedures divided on indication groups. Overall, females had better survival than males in all indications except valves. The patients caught up with the background population, although late in ACS, CCS, arrhythmias and others but not in valves and cardiac insufficiency. Odds-ratio females/males: ACS 0.89 (0.84–0.93); CCS 0.67 (0.65–0.70); Valves 0.99 (0.90–1.09); Arrhythmias 0.88 (0.82–0.94); Other 0.87 (0.79–0.96); Cardiomyopathy 0.88 (0.82–0.95).

Females had overall lower mortality at all time points, except after 5 years in patients with valvular heart disease (Table 4). The relative difference gradually declined with females being 38.9% lower than males at 30 days and 31.1% lower after 5 years. Although a comparison with the background population showed higher mortality in all diagnostic groups, except for CCS (Figure 6), females had better survival in all indications except valves 0.99 (0.90–1.09) (Figure 6).

To attenuate bias in both referral to treatment and mortality the outcome factors were adjusted with relevant factors from the WDHR in logistic regression analysis (Table 5). This did not affect the overall findings. In relation to treatment within 6 months, females’ odds ratio was only 0.32 (0.31–0.35), whether as predominant factors were previous AMI, ACS and cardiogenic shock or arrest and not least valve disease. Concerning mortality, the findings were supported with higher 1-year mortality in treated (1.13 (1.04–1.23)) and lower mortality in non-treated (0.69 (0.63–0.76)). The dominant factors regarding mortality were indication.

**Figure 7:**
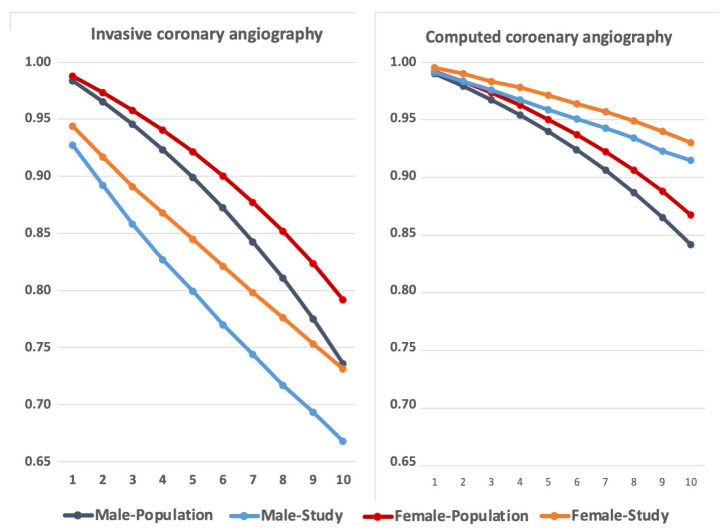
Actual and calculated population 10-year survival of all first-entry patients divided by diagnostic tool and sex. ICA study patients had overall lower survival than background while in CTA study patients had higher survival than background from start in females and after 2 years in males.

Comparison of survival related to diagnostic procedure types showed that ICA patients had lower survival, while CTA patients had better survival than the background population (Figure 7). Further, independently of procedure type, first-entry non-treated females had lower mortality than males, being 0.71 (0.69–0.73) after ICA and 0.76 (0.71–0.81) after CTA (Figure 7). The higher use of CTA with more seemingly more healthy patients during the last decade may have impacted both on referral to early treatment and mortality. However, the logistic regression analysis showed an independent impact on the lower treatment as well as on higher mortality in the treated group and on the lower mortality in non-treated group.

## 4. Discussion

We analysed periprocedural data of 274,617 first-entry patients submitted for CVD diagnostication between 2000 and 2020, concentrating on survival in relation to subsequent treatment versus diagnostication only with primary focus on sex differences. Females were treated less often than males: 28.2% vs. 49.1% within 6 months of the diagnostication procedure. In agreement with earlier findings, females had poorer survival after treatment than males at all points of time after CABG [21], aortic valve replacement [22] and PCI [8], but notably a better survival than males in the non-treated patients.

The invasive treatment fraction gradually decreased during the decades from 44.0% in the first to 23.1% in the last 5-year period, especially following the introduction of CTA in 2008. The massive increase in CTA was followed by an absolute increase in invasive treatments, but despite a factor 2.6 increase in diagnostic procedures, the fraction of non-treated patients increased from 56.0% to 76.9% during the same periods. The fall in the treatment fraction was primarily due to extensive use of CTA in women with suspected CCS, but increase in CTA indications like arrhythmias and cardiomyopathy with limited invasive treatment options may be contributing as well.

In Denmark, females account for 39% and males for 61% of IHD patients [23,24], closely matching the 40.2% and 59.8% representation among the 274,617 first-time entries in the Western Denmark Heart Registry. However, females comprised merely 27.9% of the treatment procedures, a gap that may have several explanations.

In primary care, females constitute 43.9% of consultations, a figure slightly above their IHD prevalence and thus suggesting equitable conservative referral practices in the primary sector. Consequently, the female treatment underrepresentation seems to take place after the diagnostic procedure.

The lower invasive treatment activity in females originates from relatively more healthy females being referred for investigational procedures, as non-treated CTA-diagnosticated females showed better survival than the background population (Figure 7). Contrasting with ICA-diagnosticated patients, CTA-diagnosticated patients had markedly better survival than the background population. This survival difference between ICA- and CTA-diagnosticated patient groups agrees with an earlier study on CCS patients suggesting that a high number of patients without obvious cardiac disease were referred to a CTA procedure [25].

It has been a concern that the rapidly growing use of CTA might increase the number of ICA and even PCI procedures through large numbers of falsely positive CCTA investigations [26,27,28]. This is probably not the case, as the number of ICA procedures gradually declined during the last decade, despite an increasing number of invasive treatments for ACS and valvular heart disease (Figure 3).

The fraction of diagnostic procedures resulting in interventional treatment showed huge differences between indications (Table 3). The number of patients planned for invasive treatment, where the treatment was not affected, was overall only 0.6%. The fraction was 1.0% or lower in all indications, except cardiogenic shock (1.7%) and valve disease (6.2%), indicating that few of those patients were left without treatment. The reason for the missing treatment may to some extent be due to early death, especially in patients with cardiogenic shock or cardiac arrest with a 7-day mortality of 47.6%. Other explanations for missing treatment may be high treatment risk or patients declining treatment.

In contrast to invasive treatment groups, non-treated females showed better survival than males in all indication groups (Figure 6), with the exception of heart valve disease 0.99 (0.90–1.09). These findings are supplemented by mortality comparisons with the background population (Figure 6), demonstrating that all indication groups had lower survival than the background population, with the exception of CCS patients.

Females continue to experience higher CVD-related mortality, even though their mortality rates have declined more than that of males during last decades [4,9,10]. This disparity is believed to be caused by factors such as older age, more comorbidities and risk factors, a greater incidence of procedural complications, and less frequent use of evidence-based invasive therapy [10,11,29,30]. The higher incidence of complications was demonstrated in previous studies [8,21], but in this study of patients with or without invasive treatment, females were only marginally older and with only minor differences in comorbidities, essentially leaving the less frequent invasive treatment as a major issue between the sexes. The superior survival of females in the non-treated groups might indicate that females with a potentially better survival may not have been scheduled for invasive treatment.

The poorer invasive treatment outcome in females may also be related to the more challenging anatomy of female patients, such as smaller genuine coronary arteries and conduits, a more diffuse pattern of coronary disease in addition to implantation of smaller sizes of heart valve prostheses with increased risk of patient-prosthesis-mismatch [31,32]. However, females seem less challenged because of fewer diseased coronary arteries, lower degree of coronary stenosis in treated and untreated patients (Table 2), and a smaller number of CABG conduits below 1.5 mm in diameter (28.0% vs. 29.0%; *p* = 0.047). The small differences in coronaries, which in principle favour females, cannot explain the marked disparity in revascularization rates and outcomes.

### Strengths and Limitations

A key advantage of this study is its large, representative cohort from the WDHR with more than 600,000 registered procedures of which 274,617 first-entry patients were obtained. The number augments the generalizability and trustworthiness of the findings. The crucial strengths are the mandatory and obligatory nature of prospectively reported data from a well-defined uptake area into a shared mandatory database used by all relevant institutions. The large cohort with detailed in-hospital outcome and complete mortality follow-up on all patients undergoing invasive cardiologic and cardiac surgery procedures for more than two decades allows robust estimations of patients, results, and adverse events.

Nevertheless, the study caries intrinsic limitations and especially the non-randomized nature may cover additional effects of missing covariates and potentially increase confounding. Further, the observational bearing might introduce biases and muddling factors. Although the public free health care system with uniform structure and treatment practices reduce variability, the long study period poses challenges from possible changes in clinical procedures.

## 5. Conclusions

Despite referral for coronary diagnostication in agreement with their CVD prevalence, females received less invasive treatments than males and presented with a substantially higher mortality after invasive treatments. However, non-invasive-treated females had significantly better survival than males, both in intra-study and in comparison with the background population. The massive increase in diagnostic procedures during the last decade was primarily caused by the use of CTA.

## Figures and Tables

**Table 1:** Demographics, periods and sex.

Factor	Gender	2000–2005	2006–2010	2011–2015	2016–2020	*p*-Value	All
Female fraction		33.7%	39.7%	43.0%	41.6%	<0.0001 *	40.2%
**Age**	**Male**	62.6	62.8	61.5	62.4	<0.001	62.3
	**Female**	64.7	64.3	62.8	63.8		63.7
	*p*-value	<0.001					
**IHD in family**	**Male**	32.5%	37.1%	39.5%	32.4%	0.325	35.4%
	**Female**	37.9%	44.5%	46.8%	40.3%		42.9%
	*p*-value	<0.001					
**Treatment Statins**	**Male**	34.1%	45.2%	39.1%	34.4%	<0.001	38.1%
	**Female**	34.3%	45.4%	38.9%	33.7%		38.0%
	*p*-value	<0.001					
**Treatment Hypertension**	**Male**	32.3%	46.0%	46.5%	45.6%	<0.001	43.3%
	**Female**	40.9%	51.5%	48.6%	46.3%		47.3%
	*p*-value	<0.001					
**Previous Myocardial infarction**	**Male**	24.1%	9.1%	4.0%	2.1%	<0.001	8.6%
	**Female**	16.1%	5.2%	2.0%	1.0%		4.5%
	*p*-value	<0.001					
**Treatment Diabetes**	**Male**	7.8%	11.3%	11.7%	12.0%	<0.001	10.9%
	**Female**	8.1%	9.2%	9.1%	9.4%		9.1%
	*p*-value	<0.001					
**Coronaries investigated**							
**Diagnostic only**	**Male**	2.86	2.39	2.02	1.94	<0.001	2.28
	**Female**	2.52	1.97	1.74	1.66		1.95
	*p*-value	<0.001					
**Treatment within 6 months**	**Male**	2.78	2.53	2.30	2.30	<0.001	2.49
	**Female**	2.50	2.27	2.01	2.01		2.22
	*p*-value	<0.001					
**Average stenosis**							
**Diagnostic only**	**Male**	75.8	71.5	70.6	67.4	<0.001	71.2
	**Female**	70.6	64.9	65.0	64.5		66.0
	*p*-value	<0.001					
**Treatment within 6 months**	**Male**	83.1	84.1	84.8	84.5	<0.001	84.1
	**Female**	82.0	82.5	84.3	84.2		83.1
	*p*-value	<0.001					

Demographic factors and investigated coronaries of first-entry procedure divided by period and sex. * χ^2^-test. Rest 2-way ANOVA.

**Table 2:** Demographics, indication and sex.

Factor	Gender	ACS	CCS	Valves	Cardiomyopathy	Arrhythmia	Other	Shock/Arrest	*p*-Value
Female fraction		33.0%	45.1%	39.7%	30.8%	32.6%	45.0%	26.5%	<0.0001 *
**Demographics**									
**Age**	**Male**	63.8	60.8	68.8	63.5	63.0	54.5	63.6	<0.001
	**Female**	67.6	61.7	73.2	64.8	63.6	57.2	63.8	
	*p*-value	<0.001							
**IHD in family**	**Male**	35.6%	39.3%	20.2%	26.7%	25.4%	28.3%	27.9%	<0.001
	**Female**	38.5%	48.1%	22.4%	29.6%	31.9%	35.9%	18.3%	
	*p*-value	<0.001							
**Treatment Statins**	**Male**	32.5%	43.4%	34.7%	35.7%	30.8%	25.1%	28.0%	0.403
	**Female**	36.7%	39.9%	36.6%	32.1%	28.6%	29.8%	23.7%	
	*p*-value	<0.001							
**Treatment Hypertension**	**Male**	37.5%	45.6%	49.2%	46.7%	44.2%	39.2%	42.8%	<0.001
	**Female**	47.8%	46.5%	56.0%	46.7%	44.7%	43.9%	46.0%	
	*p*-value	<0.001							
**Previous Myocardial infarction**	**Male**	11.0%	8.5%	3.6%	7.8%	7.2%	1.7%	3.8%	<0.001
	**Female**	9.0%	3.4%	2.2%	5.1%	3.3%	1.1%	0.9%	
	*p*-value	<0.001							
**Treatment Diabetes**	**Male**	10.0%	11.0%	11.9%	15.4%	9.0%	9.1%	14.5%	<0.001
	**Female**	10.8%	8.2%	11.2%	12.5%	6.8%	7.5%	13.9%	
	*p*-value	<0.001							
**Treatment after diagnostic procedure**									
**None- diagnostic only**	**Male**	20.1%	60.6%	14.4%	75.2%	69.2%	45.8%	84.0%	<0.001
	**Female**	39.9%	83.0%	18.8%	82.5%	78.3%	65.6%	92.9%	
	*p*-value	<0.001							
**Treatment within 6 months**	**Male**	78.4%	35.6%	80.7%	20.7%	27.8%	53.4%	13.1%	<0.001
	**Female**	58.7%	14.8%	76.0%	14.5%	19.7%	34.4%	5.5%	
	*p*-value	<0.001							
**Later treatment**	**Male**	1.5%	3.8%	4.9%	4.1%	3.0%	0.8%	2.9%	<0.001
	**Female**	1.4%	2.2%	5.2%	3.0%	2.0%	0.0%	1.6%	
	*p*-value	<0.001							

Demographic factors of first-entry procedure divided by indications and gender. * χ^2^-test. Rest 2-way ANOVA.

**Table 3:** Result of coronary angiography.

Consequence	Sex	ACS	CCS	Valves	Cardiomyopathy	Arrhythmia	Other	Shock/arrest	All	*p*-Value
**None**	**Male**	23.7%	43.3%	14.8%	21.9%	46.5%	56.7%	44.5%	39.1%	<0.0001
	**Female**	23.3%	51.2%	13.4%	27.3%	55.2%	59.6%	48.7%	46.5%	
**Medical treatment**	**Male**	47.7%	28.4%	14.6%	51.1%	24.9%	17.2%	41.3%	32.0%	<0.0001
	**Female**	53.9%	24.5%	16.8%	47.1%	21.1%	17.6%	39.4%	28.7%	
**Conference/new diagnostic**	**Male**	8.1%	16.1%	46.6%	11.7%	9.6%	8.2%	3.0%	14.4%	<0.0001
	**Female**	5.4%	12.6%	46.1%	9.7%	7.9%	6.4%	1.7%	11.7%	
**Treatment**	**Male**	1.3%	0.6%	6.6%	0.8%	0.4%	0.1%	1.8%	0.8%	<0.0001
	**Female**	0.8%	0.3%	5.8%	0.5%	0.2%	0.3%	1.5%	0.4%	
**No info**	**Male**	19.1%	11.6%	17.4%	14.5%	18.6%	17.8%	9.4%	13.7%	<0.0001
	**Female**	16.6%	11.4%	17.9%	15.4%	15.6%	16.1%	8.7%	12.7%	

Consequences/results of first-entry diagnostic procedures of patents without invasive treatment divided by sex and indication. Statistics: χ^2^-test.

**Table 4:** Mortality of first-entry diagnostic procedures.

Mortality		ACS	CCS	Valves	Cardiomyopathy	Arrhythmia	Other	Shock/Arrest	*p*-Value	All
**7 days**	**Male**	2.78	0.13	2.53	0.61	2.06	0.85	47.66	<0.0001	1.14
	**Female**	2.24	0.09	2.05	0.88	1.66	0.82	47.52		0.74
**30 days**	**Male**	4.67	0.27	9.02	2.81	5.48	1.48	55.05	<0.0001	1.85
	**Female**	3.44	0.16	5.65	1.57	2.57	1.28	56.27		1.13
**1-year**	**Male**	10.61	1.72	28.60	6.55	5.38	3.89	59.43	<0.0001	4.70
	**Female**	8.29	0.96	24.43	6.83	4.06	4.07	58.89		3.08
**5-years**	**Male**	24.68	8.06	16.63	125.11	12.08	12.13	67.98	<0.0001	14.09
	**Female**	21.04	4.95	28.88	59.94	11.05	11.89	65.75		9.70

Mortality of first-entry non-treated diagnostic procedures divided by indication and sex. Statistics: χ^2^-test.

**Table 5:** Scheduled to treatment and 1-year mortality in treated/non-treated patients.

Adjustment Factor	Treatment < 6-Month	Mortality 1-Year	
		Treatment < 6-Month	Diagnostication Only
Age (year)	1.03 (1.03–1.03)	1.07 (1.07–1.08)	1.07 (1.06–1.07)
IHD in family	1.19 (1.16–1.23)	0.73 (0.69–0.82)	0.74 (0.66–0.82)
Anti-lipid treatment	1.27 (1.24–1.31)	0.72 (0.66–0.79)	0.75 (0.67–0.83)
Hypertension treatment	1.01 (0.95–1.01)	1.04 (0.96–1.14)	0.90 (0.81–0.99)
Diabetes treatment	1.12 (1.07–1.17)	1.64 (1.46–1.84)	1.19 (1.70–2.21)
Previous AMI	3.14 (2.96–3.33)	1.77 (1.54–1.99)	2.89 (2.49–3.36)
Left ventricular EF	1.09 (1.06–1.12)	1.02 (0.96–1.09)	1.00 (0.93–1.08)
ACS	7.85 (7.61–8.10)	Indication reference	4.90 (4.37–5.51)
CCS	Indication reference	0.46 (0.41–0.51)	Indication reference
Arrhythmia	0.26 (0.24–0.29)	1.61 (1.18–2.18)	1.83 (1.50–2.25)
Cardiac insufficiency	0.55 (0.51–0.59)	1.64 (1.35–2.00)	3.35 (2.89–3.89)
Other	1.07 (0.98–1.16)	2.84 (2.18–3.71)	2.70 (2.11–3.44)
Cardiogenic Shock	3.65 (2.82–4.73)	8.9 (5.41–11.6)	67.7 (44.4–103)
Valve disease	15.1 (14.2–16.2)	0.81 (0.72–0.91)	11.2 (9.47–13.2)
Sex	0.32 (0.31–0.35)	1.13 (1.04–1.23)	0.69 (0.63–0.76)

Odds ratio (95% CL) of factors with possible impact on referral to treatments and 1-year mortality divided by treatment and diagnostication only. IHD = Ischaemic heart disease; ACS = Acute coronary syndrome; CCS = Chronic coronary syndrome; AMI = Acute myocardial infarction.

## Data Availability

The datasets generated for use in the current study are not publicly available as part of the Danish patient/hospital system, but limited blinded data are available from the corresponding author on reasonable request and subsequent permissions from hospital/database managers.

## Supplementary Materials

The following supporting information can be downloaded at: https://www.mdpi.com/article/10.3390/medicina62010144/s1, Table S1: First entry patient/procedures divided on indication group. Table S2: Consequences after index procedure grouped into more comparable groups. Figure S1: Population survival divided on time periods and gender.

## Abbreviations

ACS	Acute coronary syndrome
AMI	Acute myocardial infarction
AP	Stable angina pectoris
CABG	Coronary arterial bypass grafting
CCS	Chronic coronary syndrome
CTA	Computed tomography angiography
CPR	Civil personal registration
CVD	Cardiovascular disease
ICA	Invasive coronary angiography
ICD-10	International classification of diseases, 10th revision
IHD	Ischaemic heart disease
LVEF	Left ventricular ejection fraction
PCI	Percutaneous coronary intervention
TAVR	Transcatheter aortic valve replacement
WDHR	Western Denmark Heart Registry
