# Correction: Bhavsar et al. Female Disparity in Referral to Cardiac Diagnostication and Invasive Treatment. *Medicina* 2026, *62*, 144

**DOI:** 10.3390/medicina62030472

**Published:** 2026-03-02

**Authors:** Rajesh Bhavsar, Leif Thuesen, Carl-Johan Jakobsen

**Affiliations:** 1 Department of Anaesthesiology and Intensive Care, University Hospital of Southern Denmark, 6200 Aabenraa, Denmark; rbhavsar@health.sdu.dk; 2 Department of Regional Health Research, University of Southern Denmark, 5230 Odense, Denmark; 3 Department of Cardiology, Aalborg University Hospital, 9000 Aalborg, Denmark; thuesenleif@gmail.com; 4 Department of Anaesthesiology, Heart, Lung and Vessel, Aarhus University Hospital, 8200 Aarhus N, Denmark

**Error in Table**

In the original publication [1], there was a mistake in Table 4 as published. Five-year mortality is wrong due to a calculation error. The corrected Table 4 appears below. The authors state that the scientific conclusions are unaffected. This correction was approved by the Academic Editor. The original publication has also been updated.

## Figures and Tables

**Table 4:** Mortality of first-entry diagnostic procedures.

Mortality		ACS	CCS	Valves	Cardiomyopathy	Arrhythmia	Other	Shock/Arrest	*p*-Value	All
**7 days**	**Male**	2.78	0.13	2.53	0.61	2.06	0.85	47.66	<0.0001	1.14
	**Female**	2.24	0.09	2.05	0.88	1.66	0.82	47.52		0.74
**30 days**	**Male**	4.67	0.27	9.02	2.81	5.48	1.48	55.05	<0.0001	1.85
	**Female**	3.44	0.16	5.65	1.57	2.57	1.28	56.27		1.13
**1-year**	**Male**	10.61	1.72	28.60	6.55	5.38	3.89	59.43	<0.0001	4.70
	**Female**	8.29	0.96	24.43	6.83	4.06	4.07	58.89		3.08
**5-years**	**Male**	24.68	8.06	63.08	25.39	15.69	12.13	67.98	<0.0001	14.09
	**Female**	21.04	4.95	62.20	23.54	13.07	11.89	65.75		9.70

Mortality of first-entry non-treated diagnostic procedures divided by indication and sex. Statistics: χ^2^-test.

## References

[1] Bhavsar R., Thuesen L., Jakobsen C.-J. (2026). Female Disparity in Referral to Cardiac Diagnostication and Invasive Treatment. Medicina.

