# Supplementary material for: Female Disparity in Referral to Cardiac Diagnostication and Invasive Treatment
Source: Medicina (Kaunas). 2026 Jan 10;62(1):144. doi: 10.3390/medicina62010144 (PMC12843300; doi:10.3390/medicina62010144)
Supplement: Supplementary file 1 [file medicina-62-00144-s001.zip › Figure S1.pdf]

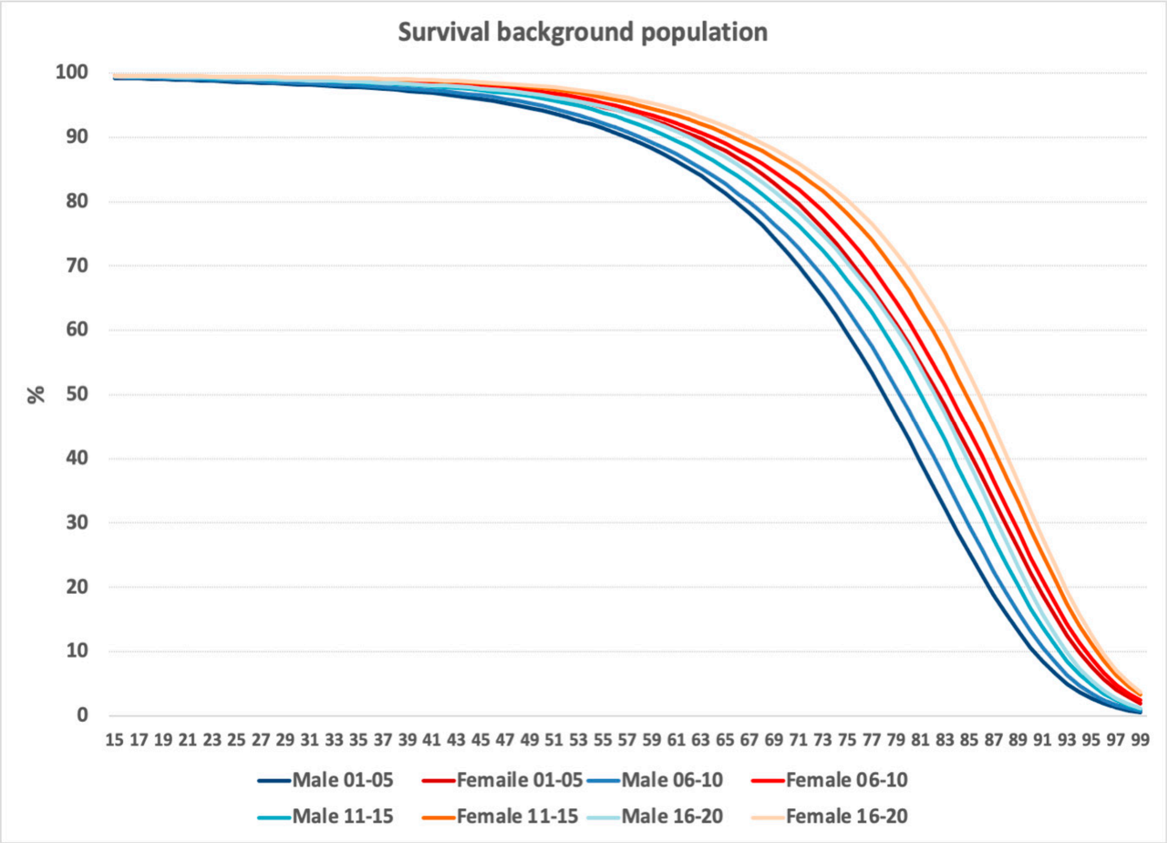

Figure S1. Population survival divided on time periods and gender. Survival curves based on 1-year mortality of the actual age (15-99 years); Data from Danish Statistics. https://www.dst.dk/en/.
