# Supplementary material for: Female Disparity in Referral to Cardiac Diagnostication and Invasive Treatment
Source: Medicina (Kaunas). 2026 Jan 10;62(1):144. doi: 10.3390/medicina62010144 (PMC12843300; doi:10.3390/medicina62010144)
Supplement: Supplementary file 1 [file medicina-62-00144-s001.zip › Table S1.pdf]

Registered indication	ACS	CCS	Valve	Cardio-myopathy	Arrhythmia	Other	Cardiogenic shock/arrest	All
**Intravascular Coronary Angiography**								
STEMI	29.729							29.729
N-STEMI / Ustable AP	39.620							39.620
Angina Pectoris - stable		59.949						59.949
Complementary/complication		121	5	9				135
Valve disease			14.668	2				14.670
Cardiomyophaty/Insufficiency	4		3	11.586			2	11.595
Arrhythmia	74	388	13	91	5.255			5.821
Unclear Chestpain		2.070	13	24	18	1.440	1	3.566
Other	376	3.328	109	288	350	2.792	59	7.302
Cardiogenic Shock / arrest							1.903	1.903
**Computed tomography angiography**								
Angina/angina-equivalent		73.782						73.782
Anatomy and grafts		9	1	1		731	0	742
Other/non specified	28	178	416	28	3.451	2.190	0	6.291
Valve and aortic disease		882	1.285	65	2.472		0	4.704
Cardiomyophaty/Insufficiency				2.140			0	2.140
**Percutaneous cardiac intervention**								
STEMI	413							413
N-STEMI / Ustable AP	1.400							1.400
Angina Pectoris - stable		3.272						3.272
Complementary/complication		5						5
Valve disease			6					6
Cardiomyophaty/Insufficiency				55				55
Arrhythmia					33			33
Other	1	194						195
Cardiogenic Shock / arrest							6	6
**Cardiac Surgery and TAVR**								
CABG		4.024						4.024
Valve Surgery			1211					1.211
CABG + Valve			450					450
Other						562		562
Aortic surgery						928		928
**TAVR**			108					108

Table S1. First entry patient/procedures divided on indication group. Group primary based on WDHR codes supplemented with ISD-10 codes in less specific groups or unclear
