# Supplementary material for: Female Disparity in Referral to Cardiac Diagnostication and Invasive Treatment
Source: Medicina (Kaunas). 2026 Jan 10;62(1):144. doi: 10.3390/medicina62010144 (PMC12843300; doi:10.3390/medicina62010144)
Supplement: Supplementary file 1 [file medicina-62-00144-s001.zip › Table S2.pdf]

Primary WDHR	Grouped	No
**Invasive coronary angiography**		
No treatment	None	22.303
Medical treatment	Medical	32.723
Surgery	Treatment	320
Conference	Conference new examination	6.303
ad hoc PCI	Treatment	128
elektiv PCI	Treatment	505
No info		14.461
Computed tomography angiography		
No treatment	None	44.107
Medical treatment	Medical	14.431
ICA	Conference new examination	6.772
PECT/SPECT	Conference new examination	3.615
FFR-CT	Conference new examination	541
Non-invasive	Conference new examination	2.752
Other	Conference new examination	138
Agatstonscoren to high - stopped		123
No info		6.085

Table S2. Consequences after index procedure grouped into more comparable groups.
